# Ex vivo cardiovascular magnetic resonance diffusion weighted imaging in congenital heart disease, an insight into the microstructures of tetralogy of Fallot, biventricular and univentricular systemic right ventricle

**DOI:** 10.1186/s12968-020-00662-8

**Published:** 2020-09-21

**Authors:** Cyril Tous, Thomas L. Gentles, Alistair A. Young, Beau P. Pontré

**Affiliations:** 1 Department of Anatomy and Medical Imaging, University of Auckland, Auckland, New Zealand; 2 Laboratory of Clinical Image Processing Le Centre de Recherche du Centre Hospitalier de l’Université de Montréal, Montréal, Canada; 3 Green Lane Paediatric and Congenital Cardiac Service, Starship Children’s Hospital, Auckland, New Zealand; 4 Department of Biomedical Engineering, King’s College London, London, UK

**Keywords:** Tetralogy of Fallot, Transposition of the great arteries, Systemic right ventricle, Diffusion tensor imaging, Congenital heart disease, Ex vivo, Microstructure, Ventricular septal defect, Levo, Dextro, Situs inversus

## Abstract

**Purpose:**

Common types of congenital heart disease exhibit a variety of structural and functional variations which may be accompanied by changes in the myocardial microstructure. We aimed to compare myocardial architecture from magnetic resonance diffusion tensor imaging (DTI) in preserved pathology specimens.

**Materials and methods:**

Pathology specimens (*n* = 24) formalin-fixed for 40.8 ± 7.9 years comprised tetralogy of Fallot (TOF, *n* = 10), dextro-transposition of great arteries (D-TGA, *n* = 8) five with ventricular septal defect (VSD), systemic right ventricle (*n* = 4), situs inversus totalis (SIT, *n* = 1) and levo-TGA (L-TGA, n = 1). Specimens were imaged using a custom spin-echo sequence and segmented automatically according to tissue volume fraction. In each specimen T1, T2, fractional anisotropy, mean diffusivity, helix angle (HA) and sheet angle (E2A) were quantified. Pathologies were compared according to their HA gradient, HA asymmetry and E2A mean value in each myocardial segment (anterior, posterior, septal and lateral walls).

**Results:**

TOF and D-TGA with VSD had decreased helix angle gradient by − 0.34°/% and remained symmetric in the septum in comparison to D-TGA without VSD. Helix angle range was decreased by 45°. It was associated with a decreased HA gradient in the right ventricular (RV) wall, i.e. predominant circumferential myocytes. The sheet angle in the septum of TOF was opposing those of the left ventricular (LV) free wall. Univentricular systemic RV had the lowest HA gradient (− 0.43°/%) and the highest HA asymmetry (75%). HA in SIT was linear, asymmetric, and reversed with a sign change at about 70% of the depth at mid-ventricle. In L-TGA with VSD, HA was asymmetric (90%) and its gradients were decreased in the septum, anterior and lateral wall.

**Conclusion:**

The organization of the myocytes as determined by DTI differs between TOF, D-TGA, L-TGA, systemic RV and SIT specimens. These differences in cardiac structure may further enlighten our understanding of cardiac function in these diverse congenital heart diseases.

## Introduction

Congenital heart disease (CHD) is the most common malformation arising during fetal development, with increasing prevalence worldwide [1]. Advances in paediatric cardiac surgery and intensive care medicine have increased survival in the younger population and there are now more adults with CHD than children [2]. These adults have a high risk of myocardial dysfunction and heart failure. Many surgical interventions are more palliative than curative, with many patients requiring multiple surgeries through their lifetime [3, 4]. Although reparative or palliative surgery will provide adequate anatomical correction, it is likely that abnormalities persist within the myocardial structure [5]. Moreover, surgeries on newborns may alter the structural growth pattern, resulting in intrinsic tissue problems and regional remodeling [6, 7], leading to differences in blood flow patterns through the heart and early heart failure.

Diffusion tensor imaging (DTI) provides quantitative information on tissue microstructure. In myocardium, myocytes are packed with collagen into sheetlets to facilitate wall thickening [8, 9]. DTI exploits the anisotripic diffusion of water in the myocardium, with the degree of signal attenuation related to the diffusion along the direction of diffusion encoding gradients [10] [11]. Determining the diffusion-related signal attenuation over a number of different diffusion encoding gradient directions allows a diffusion tensor to be constructed. The eigenvalues of the diffusion tensor characterize the mean diffusivity and the fractional anisotropy [10], with the first eigenvector aligned with the main direction of the myocytes [12–14] and the second eigenvector with the sheetlet orientation. The angle of the myocytes relative to the cardiac short axis plane is known as helix angle (HA); the angle known as E2A defines the sheetlet angle in relation to the radial direction.

Although ex vivo and in vivo myocardial structure have been investigated with DTI in a number of studies [15–19], little work has been done to date in assessing the myocardial architecture of CHD hearts. We hypothesise that the typical helical architecture is altered in CHD with a variety of pathologies. Many tertiary institutions have legacy pathology specimens used for teaching and research, which comprise a valuable resource for non-destructive imaging studies. The information obtained from CHD pathological specimens can provide insight into the characteristics of CHD in particular patient groups. We developed custom tools for imaging historical pathology specimens, many of which have short T2, and automatic evaluation of the images. We used these methods to examine regional differences in common and rare types of CHD.

## Materials and methods

### Specimens

DTI was performed on 24 historical formalin-fixed heart specimens, consisting of tetralogy of Fallot (TOF, *n* = 10), dextro transposition of the great arteries (D-TGA, *n* = 8), systemic right ventricle (RV) pathologies (*n* = 4, 2 single ventricle and two TGA with hypoplastic LV), situs inversus totalis (SIT, *n* = 1) and levo-TGA (L-TGA, *n* = 1). Of these 24 specimens, 17 had a ventricular septal defect (VSD), including all the TOF, 5 D-TGA specimens and 2 systemic RVs.

### Image acquisition

One of the main challenges in scanning historical ex vivo specimens is the low signal-to-noise ratio (SNR) due to the reduction of T2 from the formalin fixation process. In this study, the specimens were scanned on a 3 T (Skyra, Siemens Healthineers, Erlangen Germany) with a custom spin-echo sequence with monopolar diffusion encoding gradients, eliminating susceptibility artefacts and minimising echo time (TE) as compared to typical echo planar imaging (EPI)-based techniques. To preserve the integrity of the tissues, all specimens were scanned in the same formalin-filled container that was used for their long-term storage. To comply with our ethical permissions, each specimen was scanned in its jar filled with formalin and placed at isocentre and scanned with a body 18 3 T Tim coil and the 32 channel spine coil. The diffusion sequence design, the data analysis for each specimen and congenital heart diseases groups are provided on https://github.com/c-tous/cardiac-diffusion-MRI.

Imaging parameters used were: TR = 1 s, TE = 56.88 ms, *δ* = 20.67ms, *∆* = 27.09 ms, slices = 3 (at mid-ventricle, or under the VSD when occurring), slice thickness = 4 mm, matrix = 100 × 100, field of view = 200 × 200 *mm*^2^, acquisition voxel = 2x2x4 *mm*^3^, 2 averages; 32 diffusion-encoding gradients on a single half-shell calculated by dmritool [20]. Total acquisition time for each specimen was 1 h. Non-diffusion weighted images (b = 0 *s*/*mm*^2^) were acquired every ten directions to minimize the load on the gradients. The optimal highest b value required without loss of diffusion information was calculated by b = 1.11/ADC [21, 22]. Since the mean diffusivity across specimens was 1.30 ± 0.27 × 10^−3^*mm*^2^/*s*, a b-value of *b* = 853 *s*/*mm*^2^ was used.

### Definition of the radial vector

The radial vector was defined as the direction of minimal distance between epicardium and endocardium in both the left ventricle (LV) and RV. The location of each pixel between the endocardium and epicardium along the radial vector was calculated and expressed as a percentage, with 0% corresponding to the endocardium and 100% to the epicardium. The circumferential direction was defined to be perpendicular to the radial direction and the longitudinal direction was defined in the apex-base direction (out of the plane of the image).

### Helix and sheet angles

**Fig. 1:**
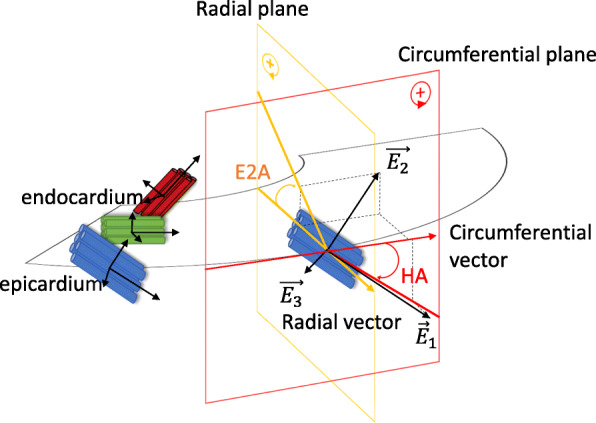
Helix angle (HA in red) and sheet angle (E2A in yellow) calculated from the projection of the eigenvectors of the diffusion tensor onto the circumferential and radial planes, respectively

Figure 1 details the calculation of the HA and E2A, respectively, according to the eigenvectors of the diffusion tensor with $\overrightarrow{E_1}$ the main eigenvector which aligns with the orientation of the myocytes [9, 12, 23, 24]. The HA [°] was defined as the angle between the projection of the first eigenvector of the diffusion tensor onto the circumferential-longitudinal plane, and the circumferential vector. The E2A [°] was defined by the angle between the projection of the second eigenvector onto the radial-longitudinal plane and the radial vector. E2A was averaged over whole segment.

**Fig. 2:**
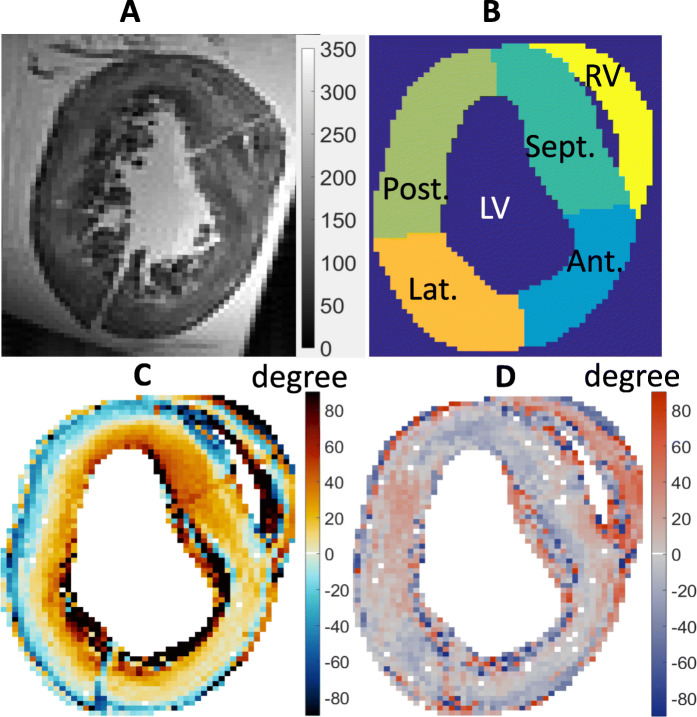
A specimen with Levo transposition of the great arteries (L-TGA). **a** spin echo diffusion weighted image at *b* = 0 *s*/*mm*^2^; **b** myocardial segment (Anterior, Septum, Posterior, Lateral walls and RV wall); **c** helix angle; **d** sheet angle. *LV*, left ventricle, *RV*, right ventricle.

The myocardium was manually segmented with papillary muscles removed from the myocardial mass. The myocardium was divided into five regions with four LV regions (anterior wall, lateral wall, posterior wall, and septum) and one region representing the RV free wall (Fig. 2).

### Helix angle gradient and asymmetry

**Fig. 3:**
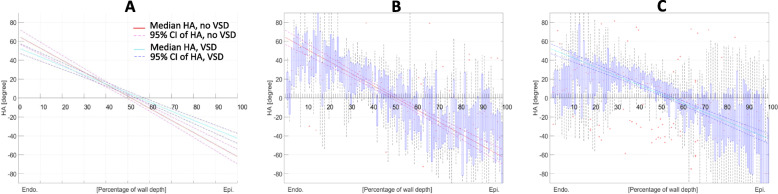
**a** Fitted line showing helix angle (HA) gradient calculated between 20 and 80% depth in biventricular dextro-tranposition of the great arteries (D-TGA) with and without ventricular septal defect (VSD). **b** Boxplot for D-TGA without VSD (*n* = 3). **c** Boxplot for D-TGA with VSD (*n* = 5). Middle line corresponds to the mean HA while outer dash lines are the 95% confident interval of the mean HA. **a** (**b**, *n* = 3) and with (**c**, *n* = 5). The Boxplot represents all HA values at each percentage of depth with the HA median as the red line, the first quartiles are the boxes and the dashed lines are the whiskers. The maximum whisker length is the interquartile range. HA values beyond the whiskers are displayed using “+”

HA values were averaged for each percentage of depth over the three acquired short axis slices acquired beneath the VSD or at the base of the ventricles in the absence of VSD. We observed that most segments showed a linear variation of HA with transmural depth over the range 20–80% depth (Fig. 3). An average HA line was fitted for each group and each segment using all points to calculate the linear gradient of the HA per degree of depth [°/%]. Endocardial HA were predominantly positive (right-handed) and epicardial angles were negative (left handed), leading to a negative gradient. Differences in slope between groups and between segments were tested using a t-test statistic incorporating error variances from each slope and weighing each of them by their degrees of freedom [25].

Oblique myocytes may not necessarily realign circumferentially at mid-wall, influencing the amount of right or left handed myocytes across the wall. Asymmetry can be computed from the intersection of the fitted line and the x axis, i.e. the depth at which the HA is zero. Hence, *HA*_0_ will be less than 50% depth for predominantly left handed myocytes (predominant epicardial fibres) and *HA*_0_ will be greater than 50% depth for predominantly right handed myocytes (predominant endocardial fibres).

## Results

### The collection of specimens

The median age at death was 8 years (quartile = [3:16], range = [1:46]) (Table 1). The median time spent in formalin was 41 years (quartile = [36:42.5], range = [29:57]). The mean T2 value over all specimens was 31.9 ± 6.5 ms and mean T1 was 146 ± 44 ms. The fractional anisotropy in the specimens was 0.24 ± 0.01.

**Table 1:** Information on the CHD specimens category, time in formalin, age of the individual at death, mean and standard deviation of T1 and T2, female(F) or male(M), the presence of VSD (Y:yes), biventricular (bi.) or univentricular (uni.) systemic right ventricle (RV), pulmonary valves stenosis (PVS) or atresia (PVA) is also specified. D-TGA = dextro-transposition of the great arteries; L-TGA – levo-transposition of the great arteries; TOF = tetralogy of Fallot;VSD = ventricular septal defect

C H D	CHD cate-gory	Years in formalin	Age	T1	T2	Sex	VSD	PVA or PVS	Surgery
1	D-TGA	29	18	180 ± 53	30 ± 6	F	Y		VSD and atrial switch
2	D-TGA	40	12	141 ± 37	34 ± 6	F	–		Atrial switch
9	D-TGA	37	15	172 ± 57	32 ± 7	F	Y	PVS	VSD and Atrial switch
10	D-TGA	41	14	117 ± 41	26 ± 8	M	Y		unoperated
11	D-TGA	43	1	165 ± 37	33 ± 4	M	–		Atrial switch
12	D-TGA	42	10	139 ± 32	27 ± 4	M	Y	PVS	Rastelli
18	D-TGA bi. Sys RV	47	6	132 ± 103	30 ± 10	M	Y	PVA	–
15	D-TGA bi. Sys RV	45	3	180 ± 48	34 ± 8	F	–		–
21	D-TGA	30	3	122 ± 36	42 ± 11	F	–		Atrial switch
22	D-TGA	37	5	161 ± 8	35 ± 8	M	Y		Arterial Switch
14	L-TGA	42	21	119 ± 14	33 ± 4	–	Y		–
3	TOF	30	34	156 ± 21	27 ± 7	M	Y	PVS	Repair
4	TOF	44	17	152 ± 40	29 ± 6	M	Y	PVS	–
5	TOF	37	1	201 ± 21	38 ± 0	M	Y	PVS	Systemic-pulmonary shunt
6	TOF	56	–	122 ± 5	22 ± 1	M	Y	PVS	Repair
7	TOF	57	17	141 ± 44	24 ± 3	M	Y	PVS	Repair
8	TOF	41	4	207 ± 47	35 ± 5	M	Y	PVS	Repair
19	TOF	50	–	69 ± 67	23 ± 4	–	Y	PVS	–
20	TOF	35	15	97 ± 43	32 ± 5	M	Y	PVS	Repair
23	TOF	32	3	153 ± 6	35 ± 7	M	Y	PVS	Repair
24	TOF	54	4	103 ± 53	26 ± 10	M	Y	PVS	Repair
13	Uni. Sys RV	42	1	178 ± 67	31 ± 5	M	–	PVA	Systemic-pulmonary shunt
17	Uni. Sys RV	31	46	183 ± 37	38 ± 6	F	–	PVA	–
16	SIT	41	1	109 ± 118	40 ± 8	F	–		–
	Max	57	46	207	42				
	Min	29	1	69	22				

### Effect of a ventricular septal defect in D-TGA

Figure 3 shows how the helix angle changes with depth through the myocardium in biventricular D-TGA specimens with and without a VSD. The HA gradient was lower when a VSD was present compared to specimens without VSD in the septum and the RV free wall. The HA range decreased from [+ 55°:-42°] without VSD to [+ 28°:-33°] with VSD. Mean E2A in the septum changed from + 17° ± 18° in non VSD to − 20° ± 49° in VSD.

### Tetralogy of Fallot and Dextro-transposition of the great arteries

**Fig. 4:**
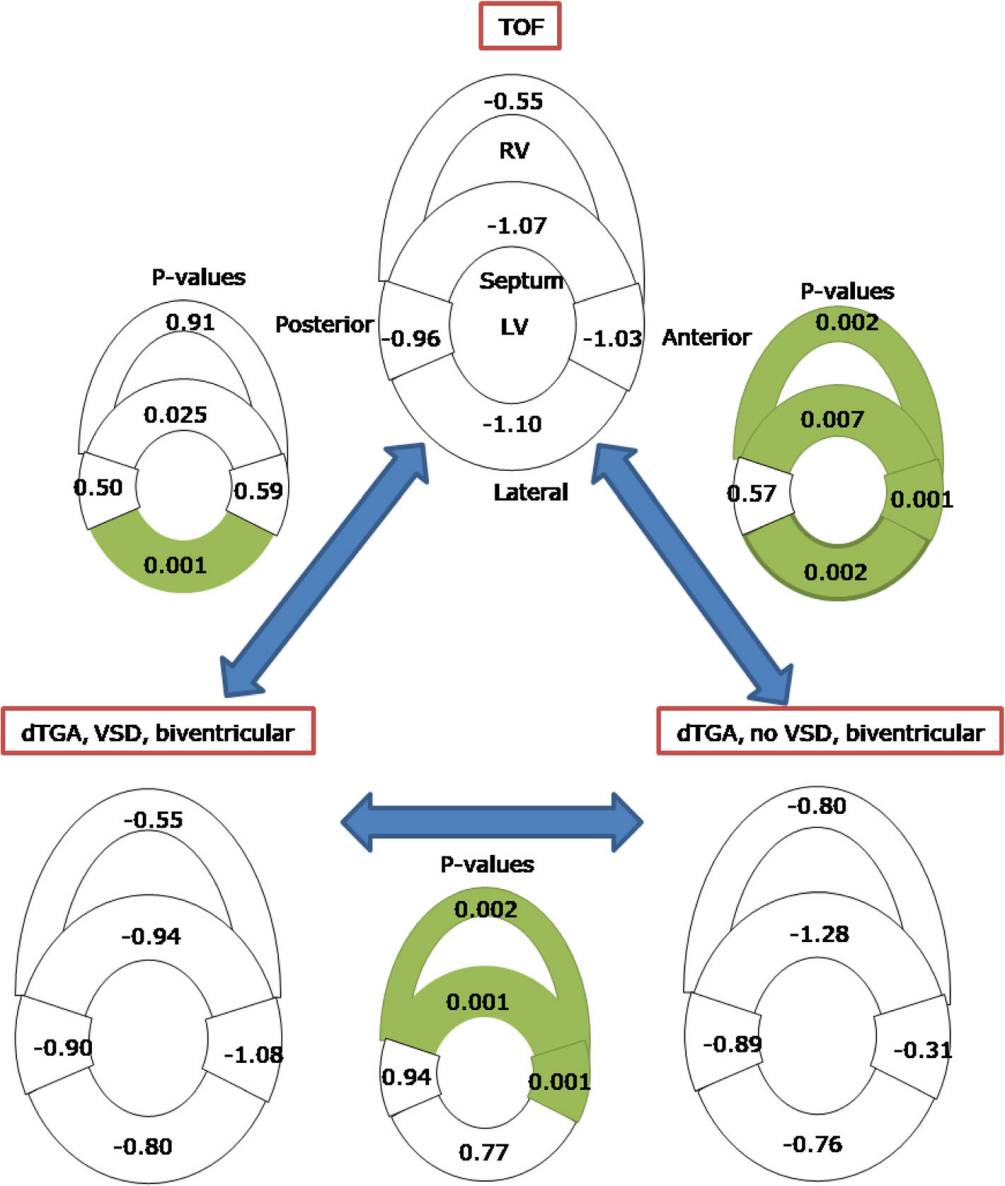
Helix angle gradient in tetralogy of Fallot (TOF), dextro-transposition of the great arteries (D-TGA) with and without ventricular septal defect (VSD). Significant differences between HA gradient (*p* < 0.01) are highlighted in green

Figure 4 shows the differences in HA gradient between the TOF group and each of the D-TGA groups (with and without VSD). In the septum, HA gradient was lower in TOF relative to D-TGA without VSD, and higher than D-TGA with VSD. In the lateral wall, HA gradient was higher in TOF than both D-TGA with VSD and D-TGA without VSD. In the RV free wall and LV anterior, the TOF group was similar to the D-TGA with VSD group but significantly lower than the D-TGA without VSD group (*p* < 0.007).

**Fig. 5:**
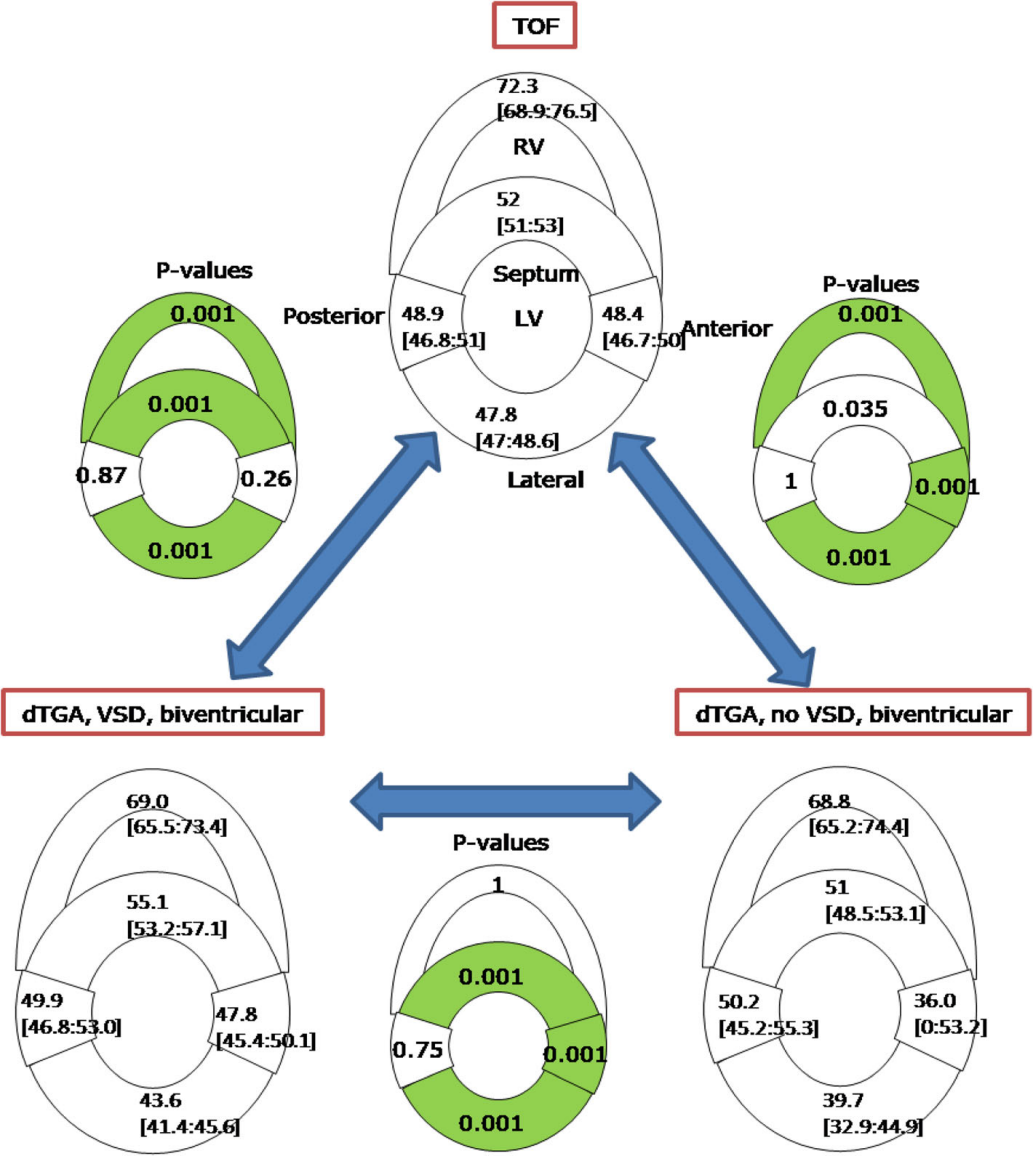
HA asymmetry (intercept of HA) with the 95% confidence interval ([lower limit: upper limit]) in specimen of tetralogy of Fallot and biventricular transposition of the great arteries with or without ventricular septal defect (VSD). The *p* value measures the significant difference between two HA intercepts from two congenital heart disease groups in each segment

The HA asymmetry in the septum of the TOF group was different from that in a bi-ventricular D-TGA with VSD (*p* < 0.001) but not from D-TGA without VSD (Fig. 5). HA asymmetry was different in RV wall of TOF and specimens with D-TGA (with or without VSD). Regardless of the pathology the HA asymmetry in the lateral wall was significantly different (*p* < 0.001).

**Fig. 6:**
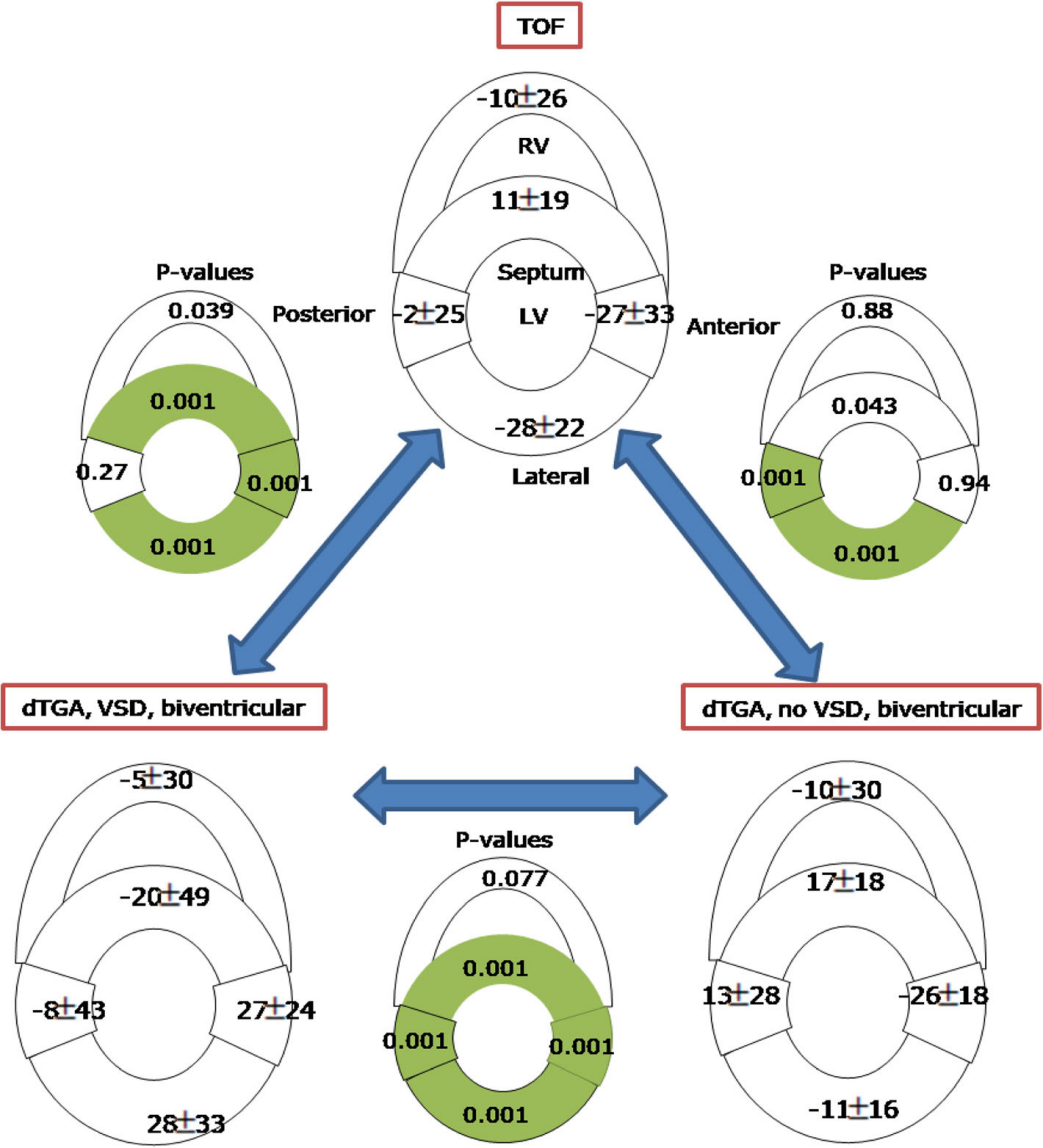
Sheet angle (E2A) in tetralogy of Fallot (TOF, *n* = 10), dextro-transposition of the great arteries (D-TGA) with and without ventricular septal defect (VSD). Significant differences (p < 0.01) are highlighted in green

The mean E2A were opposing in the septum of TOF and biventricular D-TGA with VSD (*p* < 0.001) although not in biventricular D-TGA without VSD (Fig. 6). When a VSD was present, the E2A in the septum were opposite from those of the LV free wall (*p* < 0.001). D-TGA with and without VSD had opposing E2A in the LV. RV E2A were not different across CHD pathologies.

### Systemic right ventricle

Among all CHD groups, specimens presenting univentricular systemic RV had the most decreased HA gradient (Table 2) and the highest HA asymmetry (Table 3). These asymmetries characterised dominant right-handed angles in the single ventricle. In contrast, looking at the biventricular systemic RV group, HA gradient was steeper and HA symmetry was less left handed than the single ventricles.

**Table 2:** Helix angle (HA) gradient according to the myocardial regions. Specimens number #13 and #17 make the group of univentricular systemic RV while specimens #15 and #18 make the group of biventricular systemic RV. LV = left ventricle; RV = right ventricle

HA gradient in systemic RV	Anterior	Septum	Posterior	Lateral	RV
Biventricular (LV analysis)	−0.59	−1.05	− 1.28	−1.39	− 0.98
Univentricular (RV analysis)	− 0.61	−0.43	− 0.37	−0.75

**Table 3:** Helix angle (HA) asymmetry calculated from 20 to 80% depth of the myocardial wall

HA asymmetry in systemic RV	Anterior	Septum	Posterior	Lateral	RV
Biventricular (LV analysis)	34.9 [16.0:42.3]	65.3 [60.2:74.0]	56.3 [52.4:61.6]	47.0 [43.9:49.9]	64.7 [60.9:70.2]
Univentricular (RV analysis)	74.1 [67.2:87.0]	75.1 [64.1:98.4]	68.8 [58.3:85.1]	50.8 [49:52.7]	

The group with biventricular systemic RV, in comparison to the univentricular group, had similar sheet angles with the exception of the lateral wall (*p* = 0.008) (Table 4). Within this biventricular group, the sheet angle seen in the RV wall was similar to those in the LV, while the sheet angle in the septum could be differentiated from the free wall (*p* < 0.001). The sheet angle in biventricular systemic RV was positive, which was significantly different from the negative sheet angle seen in the TOF group (*p* < 0.001).

**Table 4:** Mean sheet angle (E2A) and standard deviation calculated from 20 to 80% depth of the myocardial wall

E2A in systemic RV	Anterior	Septum	Posterior	Lateral	RV
Biventricular (LV analysis)	9 ± 14	−7 ± 26	7 ± 30	18 ± 10	6 ± 22
Univentricular (RV analysis)	13 ± 40	5 ± 35	−6 ± 25	5 ± 28	

### Situs inversus totalis

The HA gradient in the single SIT specimen was reversed (Table 5) relative to those seen in all other cases. However, HA was not linear in the region 20–80% and described a transition zone at about 70% of the depth at mid-ventricle which varied longitudinally from apex to base and according to the segment. Therefore, HA gradient was calculated within 20 to 70% depth. The HA asymmetries were segment dependent (Table 5). A scan along the longitudinal axis showed the transition from reversed helix (left-handed orientation) at the base to right-handed helix at the apex. The complete transition to normal HA appeared at about 1.2 cm from the apex and finished before segment 17.

**Table 5:** Helix angle (HA) gradient and asymmetry, as well as mean sheet angle (E2A), calculated from 20 to 70% depth of the myocardial wall in Situs Inversus Totalis

Situs inversus totalis	Anterior	Septum	Posterior	Lateral
HA gradient	0.51	0.80	0.72	0.97
HA asymmetry	74.4 [97.1:66.7]	50.4 [53.7:45.9]	53.1 [55.4:50.6]	64.4 [81.3:58.5]
mean E2A	−2 ± 34	5 ± 44	6 ± 39	−3 ± 29

### Levo-transposition of the great arteries

L-TGA with VSD had a more decreased HA gradient in the septum and anterior walls compared with the D-TGA with VSD group (Fig. 2, Table 6). In these segments, HA asymmetry was over 87% while the posterior and lateral wall remained with symmetric HA. HA asymmetry showed a rightward shift relative to D-TGA with VSD in the LV but not in the RV. The mean E2A could be statistically differentiated between dilated LV and hypertrophied RV and between the septum and the LV free wall (*p* < 0.001). E2A in the septum were more similar to D-TGA with VSD than D-TGA without VSD.

**Table 6:** Helix angle (HA) gradient and asymmetry, as well as mean sheet angle (E2A), calculated from 20 to 80% depth of the myocardial wall in L-TGA

L-TGA	Anterior	Septum	Posterior	Lateral	RV
HA gradient	−0.97	−0.98	−0.96	−0.99	−1.23
HA asymmetry	87.1 [72.1:100]	88.0 [72.2:100]	51.9 [51.3:52.5]	62.2 [59.9:64.9]	53.0 [49.1:57.7]
mean E2A	9 ± 32	−25 ± 36	−34 ± 18	5 ± 36	5 ± 26

## Discussion

Common CHD subtypes such as TOF and TGA are associated with increased prevalence of heart failure and adverse events [26–29], which may be partly associated with changes in myoarchitecture. Pathology specimens represent a valuable resource for studying the characteristics of CHD lesions. We found marked differences in cardiac microstructure associated with various CHD lesions in pathology specimens. In particular, we found that HA gradient was significantly reduced in the presence of a VSD, whether in TOF or D-TGA.

### Ventricular septal defect in TOF and D-TGA

Sanchez-Quintana [30] observed in VSD of TOF from a visual inspection that outer angles of the septum in the RV and LV are more longitudinal until the VSD where they bend circumferentially to anchor on the sides of the VSD. The measured decreased HA in our results is consistent with this qualitative description.

In healthy hearts, the HA of the septum is sigmoidal (high HA gradient) because of the dominant longitudinal myocytes across the wall [31]. From biomechanical models, an increased HA gradient is associated with more longitudinal fibres contributing to longitudinal shortening and sigmoidal HA overcomes the linear HA configurations in all measured metrics (stroke volume, ejection fraction, base and apex thickening, shortening ratio, degree of torsion) [25]. The lack of connection of longitudinal layers in the septum was also observed in pathological human fetal heart [32].

### The right ventricle in TOF and D-TGA

The presence of a VSD was also associated with a decreased HA gradient in the RV wall of TOF and D-TGA with VSD. The decreased HA gradient in the RV was not observed in the D-TGA specimens without VSD. The abnormal predominance of circumferential myocytes at mid ventricle in the RV may facilitate apical and basal dilatation, acting like a girdle on a balloon [33].

In the healthy heart, the epicardial myocytes of the RV wall wrap the LV free wall at the epicardium [32, 34] to insert in the endocardium by a crossover [35, 36]. This crossover contributes to the change of asymmetry observed in the anterior and lateral LV segment. In contrast, the endocardial myocytes of the RV merge into the septum to form the opposing oriented myocytes with the LV endocardial myocytes [31]. A change in the RV myocardial structure, such as seen in TOF and dTGA with VSD, might therefore be responsible of the changes observed in the epicardial structure of the LV at the septum, anterior and lateral walls.

### Sheet angle in TOF and D-TGA

The E2A is independent from the HA organisation [37]. In the septum of TOF, the E2A was significantly different from the rest of the LV wall (*p* < 0.001). The septum bulges toward the LV [33, 38] which may explain a change in the sheet angle. We observed that septal sheets are opposing adjacent sheets in the anterior and posterior wall. The opposite sign of the E2A between TOF and D-TGA may come from the difference of E2A in the septal bulging between TOF and D-TGA without VSD caused by the difference of ventricular pressure [39]. The difference of the E2A between myocardial segments may result from the hemodynamic change due to case-specific anatomic, vascular and post-surgical characteristics.

### Systemic right ventricle

The RV, regardless of the CHD pathology, had HA asymmetry beyond 70% (dominant endocardial orientation) with dominant circumferential myocytes across the myocardial wall (low helix angle gradient). The dominant endocardial orientation facilitates the contraction of the inner chamber at the expense of reduced epicardial lever arm force made of oblique and longitudinal orientations. Ventricular twist in RV is thus decreased and the torsion is lower, possibly increasing endocardial stress [40]. Consequently, a univentricular systemic RV has a structural disadvantage over univentricular systemic LV. The difference of HA asymmetry between univentricular and biventricular systemic RV would suggest that the absence of LV myocytes translates into the absence of LV shared parietal myocytes at the epicardium and its crossover toward the endocardium. Preserving the non-systemic ventricle during CHD surgery may maintain the orientation of the opposing myocytes necessary for efficient ventricular torsion.

In this study, univentricular systemic RV had the lowest HA gradient among all the investigated CHD groups, which is consistent with previous observations [41]. The biventricular systemic RV has previously been investigated with cardiac diffusion weighting in one patient [42]. The entire LV had an helicoidal loss with a predominance of circumferential myocytes (HA between 0 ° and 20 °) [42]. These preliminary findings are also in agreement with our measurements.

### Electrophysiology

The changes observed in myocardial microstructure of these CHD specimens may affect their electrophysiology. Electrical activation encounters less resistivity along the myocytes’ length [43–45]. There is a strong interdependence between epicardial fibre direction, conduction velocity, resistivity of the myocardium, and the potential field surrounding and generated by a wave of depolarization [45]. As a result, any changes in the helical fibre angle can affect its electrical propagation properties in charge of the cardiac function. The helicoidal fibers minimize diffusion bias of the electrical wave propagation [46]. The changes we measured in myocardial microstructure in TOF may therefore be arrhythmogenic and explain the commonly observed nonuniform and delayed polarization that is known to be associated with malignant ventricular arrhythmia and sudden death in these patients [47, 48].

### Situs Inversus Totalis and Levo transposition of the great arteries

The reversed HA in this case of SIT was in agreement with previous findings [49–53] and simulation by finite element modelling of the transition zone [54–57]. Our specimen shows the existence of the HA transition zone previously suggested in simulation to explain the reversed HA (positive HA gradient) at the base and normal HA at the apex (negative HA gradient). The transition pattern influences the sign and amplitude of systolic torsion - from positive to negative torsion [55]. The longitudinal location of these transitions can affect the systolic function [58] which may explain the inter-individual differences of torsion and gradient of circumferential radial shear in SIT. In fact, the transmural location of these HA transition pattern, specified as HA asymmetry, were observed in the helical arrangement of the basal part and vary across SIT patients [50–52], thus directly impacting the torsion pattern in the apical part.

Although a L-TGA has ventricles and valves reversed, the myocytes orientations are not reversed [52]. As previously observed in other VSD specimens, sheetlets in the septum were significantly different from the LV free wall. HA asymmetry of the LV can be a factor influencing RV output as it determines the percentage of oriented epicardial myocytes throughout the wall. The epicardial myocytes were more circumferential than longitudinal which might affect the resultant RV shortening by the LV.

The analysis of single case specimen either in L-TGA or SIT limits the conclusion about the myocytes’ orientation for this CHD group. The difference may either be due to specific defects from the individual rather than from the group or by way of noise from a single data point measurement.

### Limitations

The specimens investigated in this study comprised numerous specific and individualised diagnoses (Table S1). Further, it is possible that the histological assessment did not reference all the present pathology which may bias the interpretation of the sub-categories of defects. As a result, specimens were only categorised in broad terms according to the type of CHD, and further specific analysis of sub-categories was not considered.

The collection of pathological specimens spans many decades of changing treatment and outcome. In many of cases, the conditions were untreated, or the treatment was unsuccessful. For example, some of the cases with transposition had an atrial switch procedure but did not survive long after operation. Others succumbed from RV failure and others from pulmonary vascular obstructive disease and left heart failure. Thus, we might observe a combination of abnormal fiber orientations associated with the congenital condition and then modified by the evolving pathology. Further, the results of congenital cardiac surgery have improved significantly improved during the last five decades [3, 59]. As a result, those cases in a single category (D-TGA for example) could represent a heterogeneous group with different surgical repair techniques affecting the microstructure.

Owing to the nature of the tissue collection used in this study, preserving the integrity of the specimens was paramount. Other studies assessing myocardial fiber structure have used specific tissue preparation techniques prior to scanning, or performed histological analysis on tissues [17]. In our study, manipulating the specimens or performing any destructive procedures was not possible. As such we were not able to validate findings with histology.

The phase of the cardiac cycle affects the HA and E2A [17]. Given that we average the HA or the E2A over several specimens of the same category we expect to be more robust by getting an average estimate of the E2A regardless of the cardiac phase at fixation. This average and their respective standard deviation represent the micro-structure features specific to the CHD rather than to the cardiac phase cycle. However, with smaller number of specimens we become more subject to this cardiac fixation stage and the specific defect or noise measurements from the specimen(s).

The CHD collection investigated in our study has a disparate heart maturation which can affect the interpretation of fibre orientations as a consequence of aging rather than the pathology itself. Sanchez-Quintana [38] found that neonate hearts have circumferential epicardial helix while few weeks before birth they become oblique. Before 15 years old, the endocardial angles in the RV and LV run longitudinally and become slightly oblique to arch around the tricuspid valves. Specimens without VSD or individuals at the age of 15 years old may present more longitudinal endocardial angles than others (high HA gradient).

## Conclusion

Myocardial architecture can be non-destructively examined in specimens of CHD with CMR DTI. The differences in microstructure observed between different types of CHD specimens may explain some of the anatomical and functional observations made in CHD patients.

## Supplementary information

**Additional file 1: Table S1.** Complete diagnosis of the specimens according to their corresponding scan order (CHD number #).

## Data Availability

the datasets supporting the conclusions of this article are available in the github repository, https://github.com/c-tous/cardiac-diffusion-MRI. It contains the design of the diffusion sequences, the data analysis for each specimen and congenital heart diseases groups.

## Abbreviations

ADC: Apparent diffusion coefficient
CHD: Congenital heart disease
DTI: Diffusion tensor imaging
D-TGA: Dextro-transposition of the great arteries
E2A: Sheet angle (second eigenvector angle)
EPI: Echo planar imaging
FA: Fractional anisotropy
HA: Helix angle
L-TGA: Levo-transposition of the great arteries
LV: Left ventricle/left ventricular
RV: Right ventricle/right ventricular
SIT: Situs inversus totalis
SNR: Signal-to-noise ratio
T2: Transverse relaxation time
T1: Spin-lattice relaxation time
TE: Echo time
TGA: Transposition of the great arteries
TR: Repetition time
TOF: Tetralogy of Fallot
VSD: Ventricular septal defect
