# Supplementary material for: Ex vivo cardiovascular magnetic resonance diffusion weighted imaging in congenital heart disease, an insight into the microstructures of tetralogy of Fallot, biventricular and univentricular systemic right ventricle
Source: J Cardiovasc Magn Reson. 2020 Sep 21;22:69. doi: 10.1186/s12968-020-00662-8 (PMC7504600; doi:10.1186/s12968-020-00662-8)
Supplement: Supplementary file 1 — Additional file 1: Table S1. Complete diagnosis of the specimens according to their corresponding scan order (CHD number #). [file 12968_2020_662_MOESM1_ESM.docx]

# Supplementary Material, complete diagnosis of the specimens

R1.3

R2.4

Table S1 Complete diagnosis of the specimens according to their corresponding scan order (CHD number #)

| CHD # | CHD category | diagnosis |
| --- | --- | --- |
| 1 | TGA | Malposition of the great arteries;transposition d-loop; ventricular septal defect, subpulmonary or conal s/p VSD closure and atrial switch operation |
| 2 | TGA | Transposition d-loop with intact ventricular septum s/p atrial switch operation |
| 9 | TGA | Transposition d-loop; ventricular septal defect, subpulmonary or conal; pulmonary stenosis, subvalvar; patent ductus arteriosus s/p VSD closure and atrial switch operation |
| 10 | TGA | Transposition d-loop; ventricular septal defect, muscular; patent ductus arteriosus; pulmonary vascular occlusion (unoperated) |
| 11 | TGA | Transposition d-loop with intact ventricular septum s/p atrial switch operation |
| 12 | TGA | Transposition d-loop; ventricular septal defect, av canal type (basal); bicuspid pulmonary valve; pulmonary stenosis, valvar; pulmonary stenosis, subvalvar; right aortic arch s/p Rastelli operation |
| 18 | Biventricular Systemic RV | D-malposition of the great arteries; double outlet right ventricle; pulmonary atresia, valvar; ventricular septal defect, multiple; bilateral conus; hypoplastic left ventricle (unoperated) |
| 15 | Biventricular Systemic RV | D-malposition of the great arteries; common ventricle, comprised of both right + left; mitral atresia; double outlet right ventricle; bilateral conus (unoperated) |
| 21 | TGA | Transposition d-loop with intact ventricular septum s/p atrial switch operation |
| 22 | TGA | Transposition d-loop; ventricular septal defect, subpulmonary or conal s/p VSD closure and arterial switch operation |
| 14 | LTGA | Common inlet ventricle, left; double inlet left ventricle; transposition l-loop; coarctation; ventricular septal defect, restrictive; pulmonary vascular occlusion with cardiac shunt (unoperated) |
| 3 | TOF | Tetralogy of Fallot; bicuspid pulmonary valve; congenitally absent ductus arteriosus s/p repair without transannular patch |
| 4 | TOF | Tetralogy of Fallot; pulmonary valve abnormality; pulmonary stenosis, valvar; secundum, atrial septal defect; congenitally absent ductus arteriosus; aortopulmonary; collaterals, primitive multiple; pulmonary stenosis, peripheral, right; acute bacterial endocarditis (unoperated) |
| 5 | TOF | Tetralogy of Fallot; pulmonary valve abnormality; hypoplastic left pulmonary artery s/p aortopulmonary shunt |
| 6 | TOF | Tetralogy of Fallot s/p repair without transannular patch |
| 7 | TOF | Tetralogy of Fallot; lsvc to coronary sinus s/p repair with transannular patch |
| 8 | TOF | Tetralogy of Fallot; bicuspid pulmonary valve; secundum, atrial septal defect s/p repair with transannular patch |
| 19 | TOF | Tetralogy of Fallot; bicuspid pulmonary valve; pulmonary stenosis, valvar (unoperated) |
| 20 | TOF | Tetralogy of Fallot; lsvc to coronary sinus; familial CHD; diabetes maternal s/p repair with transannular patch |
| 23 | TOF | Tetralogy of Fallot; bicuspid pulmonary valve; lsvc to coronary sinus s/p repair with transannular patch |
| 24 | TOF | Tetralogy of Fallot; congenitally absent ductus arteriosus s/p repair without transannular patch |
| 13 | Univentricular Systemic RV | Dextrocardia; common inlet or double inlet ventricle, right; double outlet right ventricle; pulmonary atresia congenital; bilateral conus; tricuspid stenosis; large eustachian; pulmonary stenosis, peripheral, right; pulmonary stenosis, peripheral, left; hypoplastic valve; |
| 17 | Univentricular Systemic RV | Double outlet right ventricle; pulmonary atresia, valvar; pulmonary atresia, subvalvar; aortopulmonary collaterals, primitive multiple; hypoplastic main pulmonary artery; hypoplastic right pulmonary artery; hypoplastic left pulmonary artery (unoperated) |
| 16 | Single Ventricle, RV (SIT) | Situs inversus totalis; dextrocardia; malposition of the great arteries; common inlet or double inlet ventricle, right; double outlet right ventricle; complete common av canal; bilateral conus; pulmonary stenosis, valvar; pulmonary stenosis, subvalvar; (unoperated) |
